# Social inequalities in the utilization of maternal care in Bangladesh: Have they widened or narrowed in recent years?

**DOI:** 10.1186/s12939-014-0120-4

**Published:** 2014-12-10

**Authors:** Mohammad Hajizadeh, Nazmul Alam, Arijit Nandi

**Affiliations:** School of Health Administration, Faculty of Health Professions, Dalhousie University, 5161 George Street, Suite 700, Halifax, NS B3H 4R2 Canada; Centre de recherche du Centre hospitalier de l’Université de Montréal (CHUM), Montreal, Canada; Institute for Health and Social Policy & Department of Epidemiology, Biostatistics, and Occupational Health, McGill University, Montreal, Canada

**Keywords:** Social inequality, Maternal care utilization, Absolute and relative inequalities, Bangladesh

## Abstract

**Background:**

Notwithstanding the significant progress in reducing maternal mortality in recent years, social inequalities in the utilization of maternal care continue to be a challenge in Bangladesh. In this study, we aim to provide a comprehensive analysis of trends in social inequalities in utilization of antenatal care (ANC), facility based delivery (FBD), and skilled birth attendance (SBA) in Bangladesh between 1995 and 2010.

**Methods:**

Data were extracted from the five latest rounds of Bangladesh Demographic Health Surveys (BDHS). The Theil index (T) and between-group variance (BGV) were used to calculate relative and absolute disparities in the utilization of three measures (ANC, FBD, and SBA) of maternal care across six administrative regions. The relative and slope indices of inequality (RII and SII, respectively) were also used to calculate wealth- and education-based inequality in the utilization of maternal care.

**Results:**

The results of the T-index suggest that relative inequality in SBA has declined by 0.2% per year. Nevertheless, the estimated BGV demonstrated that absolute inequalities in all three measures of maternal care have increased across administrative divisions. For all three measures of maternal care, the RII and SII indicated consistent socioeconomic inequalities favouring wealthier and more educated women. The adjusted RII suggested that wealth- and education-related inequalities for ANC declined by 9% and 6%, respectively, per year during the study period. The adjusted SII, however, showed that wealth- and education-related inequalities for FBD increased by 0.6% per year.

**Conclusions:**

Although socially disadvantaged mothers increased their utilization of care relative to mothers of higher socioeconomic status, the absolute gap in utilization of care between socioeconomic groups has increased over time. Our findings indicate that wealthier and more educated women, as well as those living in urban areas, are the major users of ANC, FBD and SBA in Bangladesh. Thus, priority focus should be given to implementing and evaluating interventions that benefit women who are poorer, less educated and live in rural areas.

## Introduction

Despite major progress in reducing maternal mortality, it is unlikely that many low- and middle-income countries (LMICs) will be able to achieve their Millennium Development Goal (MDG) 5 target of reducing maternal mortality by 75-percent before 2015 [1,2]. Furthermore, evidence suggests that substantial disparities in maternal morbidity and mortality persist, with the burden among poor and marginalized populations remaining stable or even increasing in several LMICs [3-6]. Inequalities in utilization of maternal health care might partly explain these patterns [7]. Therefore, emphasis has been placed in the post-MDG period on achieving universal health coverage and ensuring that progress toward health and development goals is equitable. In other words, achieving country level targets is insufficient if progress does not reach disadvantaged populations within countries, including the less-educated, poor, as well as rural residents [5,8,9]. The United Nations Commission on Information and Accountability for Women’s and Children’s Health [10] places equity as one of the cornerstones of its accountability framework [11].

Relative to other LMICs, Bangladesh has made remarkable progress toward MDG 5. Maternal mortality was reduced from 574 deaths per 100,000 live births in 1991 to 194 deaths per 100,000 live births in 2010, a more than 60% decline in last two decades [12]. Public health policies may have contributed to this decline; for more than one decade, Bangladesh has adopted a health, nutrition, and population sector program with defined national strategies for reducing maternal mortality, focusing especially on early detection and appropriate referral of complications, and improvement of quality of care [11,13-15]. In spite of overall progress made in the maternal health sector, evidence suggests that there are pronounced social gradients in utilization of key maternal care services and maternal health outcomes in Bangladesh [14,16,17]. There are, in fact, huge geographic and socioeconomic inequalities in access to maternal health services across the country [16,18]. A study using data from the Bangladesh Demographic and Health Survey (BDHS) in 2007 reported that women from the wealthiest 10% of households were eight times more likely than women from the poorest 10% households to report at least four prenatal care visits [11]. Additionally, substantially more women from wealthier households delivered at health facilities, whereas women from poorer households often delivered with untrained traditional birth attendants (TBAs) [11,19].

Monitoring inequalities in the utilization of maternal care services is important for evaluating progress toward MDG target 5B on universal access to reproductive health. It is also critical for targeting interventions to those areas and population subgroups in greatest need. To date, a number of studies have examined utilization of maternal care in Bangladesh [11,14,17,20]. However, the previous works did not provide a detailed account of social inequalities in the utilization of maternal care across different regions and socioeconomic groups. Moreover, only a few studies examined trends in the distribution of maternal health services over time. In this study, we analyzed sixteen-year trends in inequalities in antenatal care, facility based delivery, and skilled birth attendance between rural and urban areas and across six administrative divisions (i.e., Barisal, Chittagong, Dhaka, Khulna, Rajshahi and Sylhet) in Bangladesh. Further, we investigated trends in wealth- and education- related inequalities in maternal care use over time.

## Methods

### Data

We used five latest rounds of Bangladesh Demographic and Health Surveys (BDHS) conducted between 1996/97 and 2011 as the primary source of data in this study. The BDHS is part of the worldwide Demographic and Health Surveys (DHS) program, which is designed to collect information from a nationally representative sample of households in LMICs on issues related to maternal and child health, fertility and family planning [21,22]. The DHS utilizes standardized tools and measurement techniques, a similar core set of survey questions and well-trained interviewers in order to ensure standardisation and comparability of surveys across the countries [23,24]. Based on the available information in the BDHSs, we constructed a pooled dataset containing information on the utilization of maternal health care for mothers of children born alive from 1995 to 2010 (N = 22893).

### Measures

The main outcomes of interest in the study were antenatal care (ANC), facility based delivery (FBD) and skilled birth attendant (SBA). According to the World Health Organization (WHO) recommendation, ANC is defined as having a minimum of four prenatal care visits during the pregnancy. FBD is defined as giving birth at a permanent health-facility such as health centers, hospital and private clinics. Based on the definition of the WHO [25], we measured SBA as deliveries assisted by an accredited health professional such as a midwife, doctor or nurse. We used quintiles of household wealth and maternal education (no formal schooling, primary school not completed, primary school completed, junior school completed, secondary school and above completed) to measure individual socioeconomic status (SES). We used the constructed wealth (asset) index provided in all BDHS to divide the population into wealth quintiles. The DHS uses information on households assets ownership, housing and environmental conditions to construct the wealth index [26], as suggested by Filmer and Pritchett [27]. Based on the extant literature [28-34], and available information in the BDHS, we used age at birth less than 19, age at birth more than 40 and urban region to measure adjusted socioeconomic inequality in maternal care. We selected confounder variables if they influence utilization of maternal care and were associated with our primary independent variable in the study (i.e. wealth or education).

### Statistical analysis

Our statistical approach involved two steps. First, we calculated social inequalities in the utilization of our three measures of maternal care, namely ANC, FBD and SBA, between urban and rural areas and across six administrative divisions in Bangladesh. Then, we estimated wealth- and education-based inequalities in maternal care use over time.

#### Measuring inequalities between urban and rural and across divisions

To calculate absolute and relative social inequality between urban and rural areas, we used rate ratios (RR) and rate differences (RD), respectively. As there are more than two administrative divisions in Bangladesh, the Theil index (T) was employed to estimate relative inequalities in the utilization of maternal care across divisions. The T is a widely used entropy measure of inequality and can be calculated as:

1 $$ T=\kern0.5em {\displaystyle {\sum}_{i=1}^i}\kern0.5em {s}_{ih}\left[ \ln \left({s}_{ih}/{s}_{ip}\right)\right], $$

where s_ih_ is the ith division’s share of the population’s health and s_ih_ is the ith division’s population share [35]. The T ranges from zero, indicating an equal distribution, to the natural logarithm of the *N* number of social groups, with a higher value suggesting a more unequal distribution [36]. Furthermore, we used the between-group variance (BGV) as a summary of absolute inequality across divisions. The BGV can be calculated according to the following formula:

2 $$ BGV=\kern0.5em {\sum}_{i=1}^i{p}_i{\left({y}_i-\overline{y}\right)}^2, $$

where *p*_*i*_ is division *i*’s population size (i.e., number of women who gave birth in each year), *y*_*i*_ is division *i*’s average health, and $\overline{y}$ is the average health status across divisions [35].

#### Measuring socioeconomic inequalities

Wealth- and education-related inequalities in the utilization of the three measures of maternal care were calculated using the relative index of inequality (RII) and the slope index of inequality (SII). The RII and SII are regression based measures of inequalities that take into account the distribution of maternal care service utilization across the entire socioeconomic groups [37,38] and are recommended when we want to examine inequalities across populations or time [39]. The RII is defined as the ratio between the estimated utilization of maternal care among mothers in the highest and the lowest socioeconomic groups. To estimate the RII, we first rank individuals from highest (ranked 1) to lowest (ranked 0) socioeconomic status (i.e. wealth quintile and education level). Then, the population in each socioeconomic category is assigned a fractional rank, R_*i*_, based on the midpoint of its range in the cumulative distribution of the population. For example, if the most highly educated mothers comprise 10% of the all population, the range of women in this social group is given a score of 0.05 (0.10/2), and if the second category makes up of 15% of the population, the corresponding fractional rank is 0.175 (0.10 + [0.15/2]) and so on. The RII takes a value of 0 if the utilization of maternal care is equal over education levels. A negative (positive) value of the RII indicates socioeconomic inequality in favour of higher (lower) educational levels. The RII is defined as the slope of the regression line between a group's health status and its relative rank, *R* [35,40]. There is an increased preference for estimating prevalence ratios instead of odds ratios in cross-sectional studies [41,42]; therefore, similar to previous studies [43,44], we used Poisson regression models with a robust variance in the calculation of the RII because it directly provides estimates of the prevalence ratio [41,42]. The unadjusted RII was estimated using the following Poisson model:

3 $$ lnY=\alpha +\beta R+\varepsilon, $$

where *β* is the coefficient of interest and represents the RII. In addition to reporting the unadjusted RII, we also estimated the adjusted RII after accounting for potential confounders, including age at birth (less than 19 and more than 40), rural/urban region and education or wealth quintiles depending on whether we were calculating wealth- or education-based inequality in maternal care, respectively. The adjusted RII can be estimated using the following Equation:

4 $$ lnY=\alpha +\beta R+\kern0.5em {\varSigma}_k{\delta}_k{X}_k+\varepsilon, $$

where *X*_*k*_ indexes a set of *k* confounding variables in the model. A RII value less than one indicates a positive SES gradient, where the utilization of maternal care was greater among populations of higher SES.

The SII is defined as the absolute differences in the utilization of maternal care rates between the highest SES group and the lowest. The SII can be derived from the RII and the overall mean of maternal care use according to the following formula [43]:

5 $$ SII=2\times M\times \left(RII-1\right)/RII+1, $$

where *M* represents the overall mean of maternal care use (i.e. ANC, FBD and SBA). A SII value of zero expresses perfect equality and a value further away from zero indicates greater absolute socioeconomic inequality.

We used linear regression of each inequality measure (as the dependent variable) on time (16 points corresponding to the survey year) to analyse the time trend for inequalities in the utilization of maternal care services between mothers in rural and urban areas and across six divisions. We used a weighted linear regression to estimate the time trend for wealth- and education-based inequalities in maternal care use. The inverse of the standard error of the estimates were used as weights. The estimated coefficient for the time trend (i.e. the slope of the fitted regression line) indicates a decreasing (negative value) or an increasing (positive value) trend for the inequality measure over time. The slope coefficient zero implies that there is no linear trend. The p-values are presented to indicate whether the time trend is significant.

All analyses were weighted in order to account for individual survey sample designs. Using the annual female population in Bangladesh provided by the Population Division of the United Nations [45] we applied the de-normalization of standard weights approach (as per the DHS Sampling and Household Listing Manual [46]) in order to calculate an appropriate weight for each observation in the analyses. All statistical analyses were performed using version 13 of the STATA software package (Stata Corp, College Station, Tex).

## Results

### Descriptive statistics

**Figure 1:**
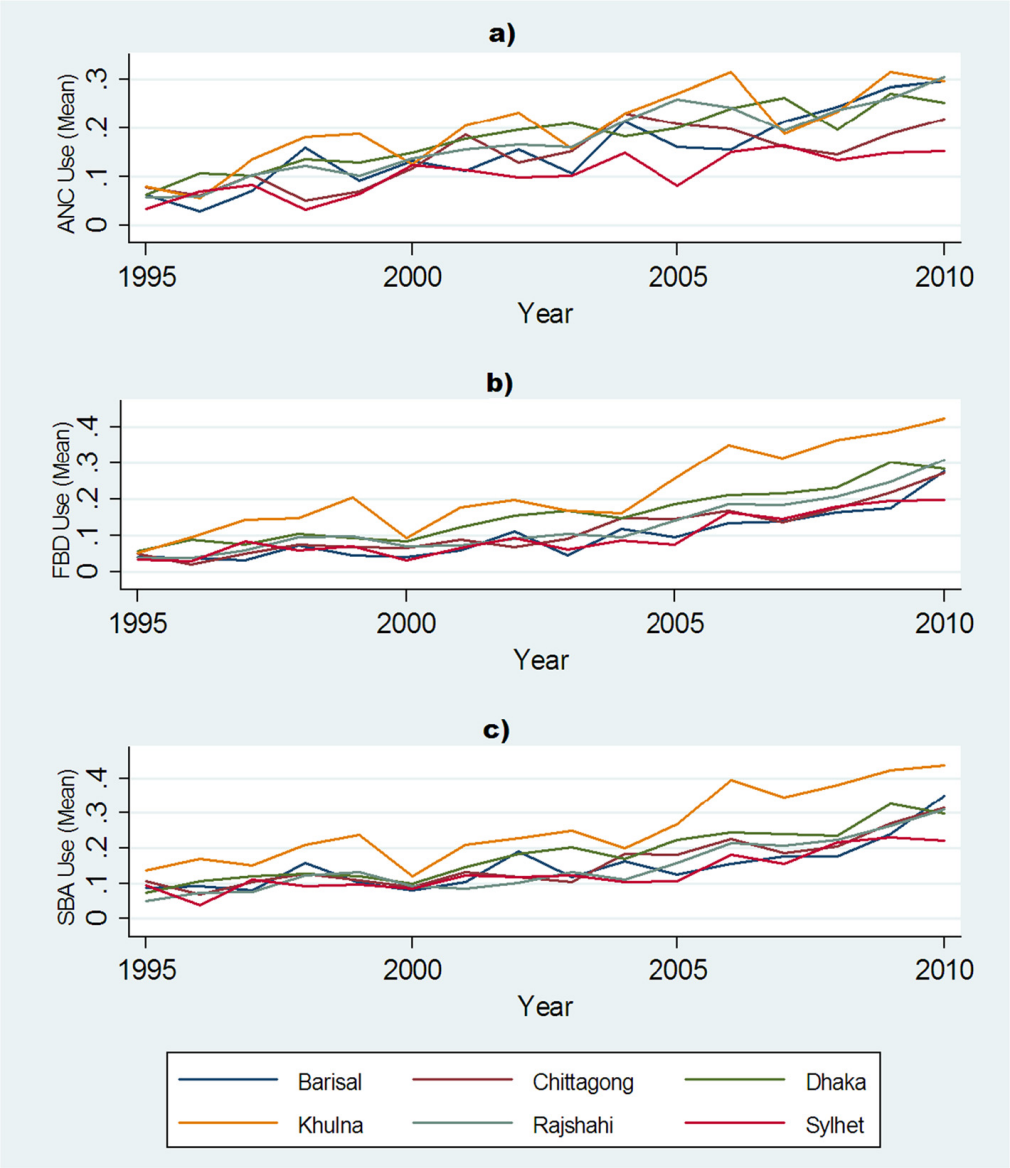
**Utilization of maternal care across six divisions of Bangladesh: 1995–2010.**

Table 1 reports the survey year, sample size and average utilization rates for ANC, FBD and SBA for the overall population as well as in rural and urban areas separately for each year. The utilization of maternal care increased according to all three measures of maternal care between 1995 and 2010. While only 6, 5 and 9 percent of women who gave birth in 1995 used ANC, FBD and SBA, respectively, these figures increased to 25, 29 and 31 percent in 2010. A steady increase in utilization of maternal care services was also observed in rural and urban areas in Bangladesh. However, there were wide differentials between urban and rural areas in the utilization of maternal care. Figure 1 shows that ANC, FBD and SBA increased in all six administrative divisions of Bangladesh (see Figure 1, Panel a, b and c).

**Table 1:** **Survey year, sample size, and maternal care use (mean) in Bangladesh: 1995-2010**

**Year**	**Survey year**	**Sample size**	**ANC**			**FBD**			**SBA**		
			**Total**	**Urban**	**Rural**	**Total**	**Urban**	**Rural**	**Total**	**Urban**	**Rural**
**1995**	1996-1997	1191	0.06	0.30	0.04	0.05	0.23	0.03	0.09	0.32	0.07
**1996**	1996-1997	1222	0.07	0.32	0.04	0.05	0.26	0.03	0.09	0.39	0.05
**1997**	1999-2000	1383	0.10	0.33	0.05	0.07	0.25	0.04	0.10	0.31	0.07
**1998**	1999-2000	1443	0.11	0.29	0.08	0.10	0.29	0.06	0.13	0.36	0.09
**1999**	1999-2000	1341	0.11	0.31	0.07	0.09	0.27	0.06	0.13	0.33	0.09
**2000**	2004	1412	0.14	0.30	0.09	0.07	0.21	0.04	0.09	0.26	0.05
**2001**	2004	1449	0.17	0.36	0.11	0.10	0.22	0.07	0.13	0.29	0.09
**2002**	2004	1361	0.17	0.36	0.12	0.12	0.29	0.07	0.15	0.37	0.10
**2003**	2004	1396	0.16	0.36	0.12	0.12	0.27	0.09	0.16	0.33	0.12
**2004**	2007	1264	0.20	0.36	0.16	0.13	0.30	0.08	0.16	0.35	0.10
**2005**	2007	1252	0.21	0.35	0.17	0.16	0.29	0.13	0.19	0.35	0.14
**2006**	2007	1201	0.22	0.44	0.17	0.20	0.39	0.15	0.24	0.43	0.18
**2007**	2011	1885	0.21	0.42	0.14	0.19	0.39	0.13	0.22	0.45	0.15
**2008**	2011	1827	0.20	0.39	0.14	0.22	0.43	0.16	0.24	0.46	0.17
**2009**	2011	1604	0.25	0.42	0.19	0.26	0.47	0.20	0.30	0.52	0.22
**2010**	2011	1662	0.25	0.48	0.19	0.29	0.53	0.23	0.31	0.56	0.24

ANC = Antenatal care, FBD = Facility based delivery, SBA = Skilled birth attendance.

**Figure 2:**
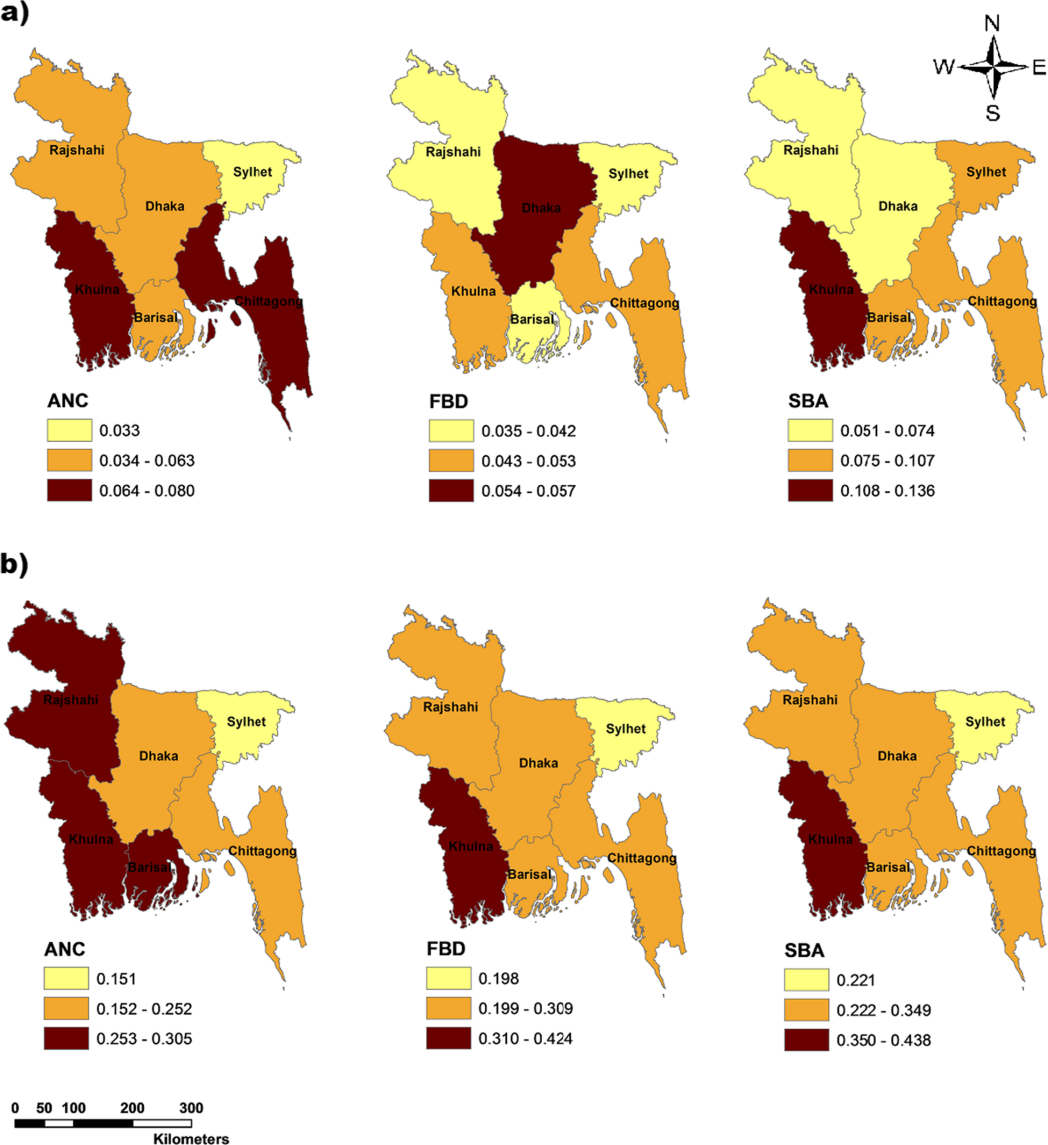
**Geographic variation in the utilization of maternal health care services in Bangladesh: a) 1995 and b) 2010.** *Note:* The Jenks natural breaks classification method was used to categorize data values into three different classes in all figures.

As illustrated in Figure 2 (Panel a, b and c), there were geographic differences in maternal care use across divisions. While the average rates of ANC were higher in Khulna and Chittagong compared to other division in 1995, women in Rajshahi, Khulna and Barisal utilized relatively more ANC in 2010 (Figure 2a). The utilization of FBD was highest in Dhaka at the beginning of the study period; however, by 2010, Khulna had the highest rate of FBD (Figure 2b). The SBA rate was higher in Khulna throughout all years. In general, the utilization of all three measures of maternal care was lower in Sylhet relative to other five divisions (Figure 2c).

### Disparities in maternal care use between rural and urban areas and across divisions

Table 2 reports inequalities in maternal care use between rural and urban areas and across six divisions in Bangladesh over the study period. It is evident that urban residents utilized more maternal care services compared to their rural counterparts for all indicators and in all years, as shown by the estimated urban–rural rate ratios. However, the relative advantage of urban women in maternal care use decreased from 1995 (RR = 6.884) to 2010 (RR = 2.526). Based on the time trend coefficients reported in the final row of the table, the magnitude of the urban–rural rate ratios for ANC, FBD and SBA declined by 0.29, 0.37 and 0.27 points per year, respectively. Similarly, urban–rural rate differences showed that women in urban areas utilized more maternal care compared to rural women counterparts. In contrast to the RR results, the RDs indicated that absolute inequalities in maternal care use between urban and rural areas increased over the study period. The increasing trend in inequality in FBD use was statistically significant, with the estimated coefficient indicating that the gap in the utilization of FBD between urban and rural areas increased by 0.5 percent per year over the period between 1995 and 2010.

**Table 2:** **Inequalities in maternal care use between rural and urban areas and across divisions in Bangladesh: 1995-2010**

	**Inequalities between urban and rural areas**						**Inequalities across divisions**					
**Year**	**RR**			**RD**			**T**			**BGV**		
	**ANC**	**FBD**	**SBA**	**ANC**	**FBD**	**SBA**	**ANC**	**FBD**	**SBA**	**ANC**	**FBD**	**SBA**
**1995**	6.884	6.998	4.842	0.257	0.196	0.250	0.019	0.013	0.048	554	213	2580
**1996**	7.336	9.224	7.144	0.277	0.236	0.337	0.055	0.168	0.057	2042	3374	3503
**1997**	6.195	6.775	4.695	0.277	0.209	0.243	0.007	0.066	0.023	705	2638	1859
**1998**	3.707	5.174	4.145	0.208	0.231	0.271	0.091	0.029	0.023	7876	1992	3223
**1999**	4.574	4.443	3.608	0.243	0.211	0.238	0.053	0.069	0.035	4602	5302	4876
**2000**	3.300	5.486	5.032	0.207	0.169	0.212	0.002	0.030	0.005	526	990	337
**2001**	3.171	3.133	3.120	0.246	0.151	0.196	0.008	0.055	0.033	2584	4294	4211
**2002**	2.960	3.982	3.818	0.237	0.220	0.270	0.022	0.065	0.044	5018	6404	6938
**2003**	3.006	3.124	2.728	0.239	0.183	0.207	0.023	0.067	0.050	4252	6372	8854
**2004**	2.311	3.629	3.468	0.205	0.219	0.251	0.005	0.023	0.023	2241	2536	3677
**2005**	2.060	2.294	2.449	0.180	0.163	0.210	0.029	0.042	0.030	8257	7186	6941
**2006**	2.584	2.574	2.353	0.268	0.236	0.249	0.017	0.029	0.024	5724	8458	9525
**2007**	2.959	2.900	2.941	0.276	0.253	0.296	0.003	0.033	0.022	5420	8143	7292
**2008**	2.773	2.715	2.652	0.251	0.273	0.285	0.005	0.026	0.019	4804	8763	7697
**2009**	2.268	2.411	2.299	0.236	0.275	0.292	0.015	0.023	0.015	7447	10383	8560
**2010**	2.526	2.312	2.302	0.291	0.298	0.319	0.016	0.014	0.011	6278	7716	7024
**Time trend coefficients**	**−0.292**	**−0.371**	**−0.23**	0.000	**0.005**	0.002	−0.002	−0.004	**−0.002**	**329**	**550**	**440**
**(P-value)**	**(0.000)**	**(0.000)**	**(0.000)**	(0.858)	**(0.031)**	(0.41)	(0.091)	(0.059)	**(0.030)**	**(0.013)**	**(0.000)**	**(0.001)**

RR = Rate Ratio, RD = Rate Difference, T = Theil Index, BGV = Between Group Variance ANC = Antenatal care, FBD = Facility based delivery, SBA = Skilled birth attendance.

Turning to inequalities in the utilization of maternal care across divisions, both the T and BGV measures indicated that ANC, FBD and SBA were not distributed equally (see the positive values for these two measures in Table 2). Although the T-index suggested that relative inequalities in SBA across administrative divisions declined by 0.2% per year, the BGV demonstrated an increasing trend in absolute inequality in the utilization of ANC, FBD and SBA between-divisions in Bangladesh.

### Socioeconomic inequalities in maternal care

Overall, results demonstrated consistent wealth- and education-related inequalities in utilization of maternal health care during the study period (Tables 3 and 4). Table 3 shows the calculated crude and adjusted RII and SII for the three measures of maternal care when we used wealth quintiles as a measure of SES. There was a positive wealth gradient in the utilization of maternal care in Bangladesh over time; specifically, women from poorer households utilized fewer maternal care services compared to those from wealthier households.

**Table 3:** **Wealth-related inequalities in maternal care use in Bangladesh: 1995-2010**

	**RII**						**SII**					
**Year**	**ANC**		**FBD**		**SBA**		**ANC**		**FBD**		**SBA**	
	**Crude**	**Adjusted**	**Crude**	**Adjusted**	**Crude**	**Adjusted**	**Crude**	**Adjusted**	**Crude**	**Adjusted**	**Crude**	**Adjusted**
**1995**	−5.489	−2.897	−5.011	−2.506	−4.436	−2.644	−0.089	−0.063	−0.065	−0.042	−0.108	−0.077
**1996**	−4.320	−2.113	−3.636	−0.273	−3.979	−1.502	−0.089	−0.051	−0.060	0.060	−0.106	−0.036
**1997**	−4.912	−2.303	−5.494	−2.793	−3.965	−1.885	−0.135	−0.080	−0.097	−0.066	−0.125	−0.064
**1998**	−3.736	−1.732	−3.355	−1.409	−3.106	−1.391	−0.131	−0.061	−0.104	−0.033	−0.137	−0.044
**1999**	−4.452	−2.511	−4.409	−2.630	−3.674	−2.279	−0.135	−0.092	−0.119	−0.085	−0.147	−0.100
**2000**	−3.497	−1.804	−3.347	−0.907	−3.123	−0.982	−0.150	−0.078	−0.077	0.007	−0.098	0.002
**2001**	−3.811	−2.090	−4.248	−2.595	−3.864	−2.233	−0.198	−0.120	−0.127	−0.091	−0.157	−0.102
**2002**	−3.039	−1.780	−4.186	−2.303	−3.921	−2.438	−0.170	−0.094	−0.144	−0.093	−0.178	−0.125
**2003**	−3.210	−1.543	−3.803	−1.980	−3.429	−1.857	−0.173	−0.070	−0.141	−0.079	−0.174	−0.095
**2004**	−2.066	−0.732	−3.248	−1.357	−3.294	−1.598	−0.141	0.063	−0.138	−0.039	−0.166	−0.072
**2005**	−2.287	−1.060	−3.478	−2.161	−3.418	−2.393	−0.164	−0.012	−0.177	−0.118	−0.207	−0.155
**2006**	−2.865	−1.635	−2.977	−1.652	−2.942	−1.935	−0.217	−0.108	−0.197	−0.098	−0.232	−0.150
**2007**	−2.833	−1.185	−3.003	−1.551	−2.978	−1.517	−0.200	−0.035	−0.189	−0.082	−0.217	−0.090
**2008**	−2.713	−1.369	−2.913	−1.776	−2.779	−1.591	−0.185	−0.062	−0.214	−0.122	−0.222	−0.107
**2009**	−2.065	−1.073	−2.374	−1.189	−2.318	−1.183	−0.171	−0.017	−0.213	−0.045	−0.235	−0.050
**2010**	−2.237	−1.233	−2.286	−1.339	−2.209	−1.253	−0.195	−0.053	−0.229	−0.085	−0.237	−0.071
**Time Trend Coefficients**	**0.177**	**0.092**	**0.152**	0.0385	**0.116**	0.044	**−0.006**	.0024	**−0.011**	**−0.006**	**−0.010**	−0.003
**(P-value)**	**(0.000)**	**(0.000)**	**(0.000)**	(0.301)	**(0.000)**	(0.073)	**(0.000)**	(0.123)	**(0.000)**	**(0.037)**	**(0.000)**	(0.099)

RII = Relative Index of Inequality, SII = Slope Index of Inequality, ANC = Antenatal care, FBD = Facility based delivery, SBA = Skilled birth attendance.

**Table 4:** **Education-related inequalities in maternal care use in Bangladesh: 1995-2010**

	**RII**						**SII**					
**Year**	**ANC**		**FBD**		**SBA**		**ANC**		**FBD**		**SBA**	
	**Crude**	**Adjusted**	**Crude**	**Adjusted**	**Crude**	**Adjusted**	**Crude**	**Adjusted**	**Crude**	**Adjusted**	**Crude**	**Adjusted**
**1995**	−4.597	−2.650	−4.455	−2.735	−3.693	−2.151	−0.083	−0.058	−0.062	−0.045	−0.098	−0.062
**1996**	−3.790	−1.994	−4.812	−3.450	−4.101	−2.519	−0.083	−0.048	−0.069	−0.058	−0.108	−0.077
**1997**	−4.365	−2.360	−4.696	−2.509	−3.559	−1.941	−0.128	−0.082	−0.091	−0.060	−0.118	−0.067
**1998**	−3.644	−2.179	−2.680	−1.240	−2.693	−1.406	−0.129	−0.084	−0.088	−0.021	−0.123	−0.045
**1999**	−3.780	−1.872	−3.642	−1.736	−2.994	−1.382	−0.124	−0.065	−0.108	−0.051	−0.128	−0.041
**2000**	−3.397	−2.228	−3.613	−2.531	−3.128	−2.114	−0.147	−0.103	−0.081	−0.062	−0.098	−0.068
**2001**	−3.374	−1.996	−3.678	−2.089	−3.464	−2.050	−0.184	−0.113	−0.117	−0.072	−0.147	−0.092
**2002**	−2.852	−1.657	−4.127	−2.746	−3.234	−1.709	−0.161	−0.083	−0.143	−0.109	−0.158	−0.078
**2003**	−3.299	−2.259	−3.996	−2.652	−3.573	−2.437	−0.176	−0.127	−0.145	−0.109	−0.179	−0.133
**2004**	−2.551	−2.011	−3.543	−2.652	−3.343	−2.398	−0.177	−0.136	−0.146	−0.118	−0.168	−0.128
**2005**	−2.634	−1.962	−3.624	−2.411	−2.952	−1.667	−0.188	−0.136	−0.182	−0.133	−0.187	−0.095
**2006**	−2.895	−1.954	−3.186	−2.253	−2.749	−1.783	−0.219	−0.145	−0.207	−0.153	−0.220	−0.132
**2007**	−2.861	−1.897	−3.016	−1.910	−2.979	−1.902	−0.201	−0.129	−0.190	−0.118	−0.217	−0.136
**2008**	−2.673	−1.753	−2.667	−1.635	−2.675	−1.727	−0.182	−0.109	−0.199	−0.106	−0.215	−0.126
**2009**	−2.118	−1.389	−2.525	−1.716	−2.477	−1.718	−0.176	−0.080	−0.227	−0.138	−0.251	−0.156
**2010**	−2.014	−1.155	−2.116	−1.257	−2.061	−1.244	−0.171	−0.037	−0.210	−0.067	−0.218	−0.069
**Time trend coefficients**	**0.140**	**0.060**	**0.132**	0.064	**0.085**	0.030	**−0.007**	−0.003	**−0.011**	**−0.006**	**−0.010**	**−0.005**
**(P-value)**	**(0.000)**	**(0.001)**	**(0.001)**	(0.069)	**(0.001)**	0.148	**(0.000)**	(0.176)	**(0.000)**	**(0.002)**	**(0.000)**	**(0.005)**

RII = Relative Index of Inequality, SII = Slope Index of Inequality, ANC = Antenatal care, FBD = Facility based delivery, SBA = Skilled birth attendance.

Based on the crude inequality measures, it is apparent that relative wealth-based inequalities in ANC, FBD and SBA declined during the study period (based on the positive values of the time trend coefficients for the crude RII), whereas absolute wealth-based inequalities increased (based on the negative values of the time trend coefficients for the crude SII). Although adjusting the relative and slope indices for confounding factors decreased the magnitudes of wealth-related inequalities, the adjusted measures, similarly to the crude RII and SII, indicated wealth-related inequalities in the distribution of maternal care favouring women from wealthier households in all study years. For example, there was a decreasing trend in the adjusted RII for ANC of 9.2 percent per year during the study period. The adjusted SII for FBD, however, increased significantly from 1995 to 2010, by 0.6 percent per year.

Education-related inequalities in maternal care use are shown in Table 4. In general, crude and adjusted values of the RII and SII suggested that more highly educated mothers in Bangladesh utilized more maternal care. Similar to findings for wealth-based inequalities, the crude RII suggested that relative education-related inequalities in the utilization of all the three types of maternal care decreased significantly from 1995 to 2010, whereas absolute education-related inequalities in maternal care in Bangladesh increased. Trends in education-related inequality in maternal care persisted after adjusting for confounding covariates. The adjusted RII indicated that education-related inequalities for ANC declined by 6 per cent per year over the study period. By contrast, the adjusted SII suggested an increase in education-based inequalities in the utilization of FBD and SBA by 0.6 percent and 0.5 percent per year, respectively (see the time trend coefficients in the table).

## Discussion and conclusions

In this study, we examined social inequalities in maternal health care utilization in Bangladesh over sixteen years. Our findings indicated that wealthier and more educated mothers utilized ANC, FBD and SBA services to a greater extent than socioeconomically disadvantaged women. Similar to other work in Bangladesh and elsewhere [4,14,47-53], our study highlighted that women living in urban areas utilized more maternal health services compared to women residing in rural regions. Although rural women increased their utilization over time, the absolute gap in the utilization of FBD between urban and rural areas increased over the period between 1995 and 2010. We also found that there was significant variation in utilisation across administrative divisions in Bangladesh, with Khulna having the highest utilization rates and Sylhet the lowest. The results of the T-index suggested that there is a trend of decreasing relative inequality across the administrative divisions for SBA over time. The estimated BGV, however, demonstrated an increasing trend in absolute inequality in the utilization of all the three measures of maternal care across administrative divisions.

Both the RII and SII suggested consistent socioeconomic inequalities in the utilization of all three measures of maternal care favouring wealthier and more educated women. These findings are consistent with earlier work [8,14] that demonstrated persistent socioeconomic inequalities in the utilization of maternal care services in Bangladesh. The adjusted SII showed that wealth- and education-related inequalities for FBD increased over the study period. The adjusted RII, however, suggested that wealth- and education-related inequalities for antenatal care declined significantly from 1995 to 2010. Thus, although poorer mothers utilized proportionally more maternal care over time, absolute differences in utilization between socioeconomic groups widened. Together these findings indicate that wealthier and more educated women are the main users of maternal care in Bangladesh.

Less utilization of antenatal care among rural residents and poorer women may result in missed opportunities to receive critical interventions for improving infant and maternal health outcomes [54]. Pregnant women receiving ANC (i.e. at least four prenatal visits) are more likely to receive preventive care and effective health promotion interventions that may be vital for the health and wellbeing of their infants [55]. Wealthier and more educated women therefore might also receive more regular examinations such as measurement of blood pressure and body weight, urine test and medicinal iron supplementation. Additionally, lack of ANC increases the risk of maternal health conditions not being diagnosed and addressed at the early stage, before they become potentially serious and life-threatening [11].

Home deliveries by untrained TBAs increase the risk for adverse maternal and infant health outcomes [56,57]. Greater availability of SBA, birthing facilities and relevant specialists is considered one of the main elements of successful *safe motherhood* interventions [58]. These conditions facilitate the timely delivery of emergency obstetric and newborn care if life-threatening complications occur. In Bangladesh several attempts have been made to improve facilities for emergency obstetric care in recent years but, nevertheless, low rates of FBD and SBA remain major challenges for improving maternal health. Most deliveries occur at home under the supervision of unskilled providers or TBAs who do not receive formal training [59]. There are also pronounced social inequalities in the distribution of FBD and SBA in Bangladesh favouring wealthy, better-educated, urban women. Our results demonstrated that socioeconomic inequalities in FBD and SBA are generally greater compared to the observed wealth- and education-related inequalities for ANC (see the crude/adjusted RII and SII in Tables 3 and 4). Thus, additional efforts are needed to promote utilization of FBD and SBA, particularly among socioeconomically disadvantaged women in Bangladesh

Several strategies both from the supply side and demand-side domain have been suggested to improve equity in uptake of maternal health care services in low-income countries. Supply-side interventions, including mobilizing health services providers at health facility and community level, enhanced monitoring and supervision, and investing in logistic and infrastructure development, may be effective [60]. Strengthening of the primary health centre network and provision of 24/7 delivery services are known to improve utilization of institutional deliveries in disadvantaged areas in rural India [61]. In Bangladesh, mobilizing services through community health care workers improved equity in utilization of maternal care services [62]. However, the demand-side barriers (e.g., user fees and lack of information, lack of transportation) to accessing maternal health services that are faced by poor women and those living in rural and remote areas in low income countries should also be addressed. Health care financing programs (e.g. free or subsidized care, cash transfer and voucher schemes) have been reported to have a positive impact on inequalities in utilization of institutional deliveries, antenatal and postnatal care and skilled birth attendants in low-income countries [61,63-74]. For example, a study by Ahmed and colleagues [69] demonstrated positive outcomes of Bangladesh maternal voucher scheme (BMVS) on inequalities in the utilization of maternal care services. The poor and vulnerable segments of community, however, typically benefit less from public health spending than their wealthier counterparts, and this must considered when implementing any program [75]. Failure to consider social and cultural factors on health care decision-making may also explain poor utilization of available services.

There were limitations to this study. First, we used information on the utilization of maternal health care for mothers during their pregnancy and delivery between two to four years prior to the survey, but our assessment of SES is based on the household wealth index constructed for the survey year. Nevertheless, changes in household wealth, in general, occur in the long-run; thus we considered the current measure of wealth to be an acceptable proxy for recent years. Second, the available sample size for each year did not allow us to examine socioeconomic inequalities in utilization of maternal care services in each administrative division separately. Third, maternal health care in Bangladesh is delivered through a community-based approach [76] and, therefore, it would have been useful to examine trends of social inequalities in utilization of community-based skilled birth attendants (CBSBA) for home deliveries. These analyses were not performed because consistent information is not available regarding the CBSBA in all of the available BDHSs.

Although Bangladesh made commendable improvements in uptake of maternal care services over the period between 1995 and 2010, our results demonstrated that the very low utilization of ANC, FBD and SBA among poorer and less-educated women, as well as those living in rural areas, remains a major impediment to further improvement in reproductive, maternal and child health outcomes. Thus, priority focus should be given on implementing and evaluating multi-sectoral interventions to improve access to and quality of care for women who are poorer, less-educated and live in rural areas in the post-MDG health and development agenda for achieving universal health coverage, as there is considerable potential for improvement among these groups [77].
